# Substrates of Sudden Cardiac Death in Hypertrophic Cardiomyopathy

**DOI:** 10.3390/jcm14041331

**Published:** 2025-02-17

**Authors:** Matteo Sclafani, Giulio Falasconi, Giacomo Tini, Beatrice Musumeci, Diego Penela, Andrea Saglietto, Luca Arcari, Chiara Bucciarelli-Ducci, Emanuele Barbato, Antonio Berruezo, Pietro Francia

**Affiliations:** 1 Royal Brompton and Harefield Hospitals, Guy’s and St Thomas’ NHS Foundation Trust, London SW3 6PY, UK; matteo.sclafani@uniroma1.it (M.S.); c.bucciarelli-ducci@rbht.nhs.uk (C.B.-D.); 2 Cardiology Unit, Department of Clinical and Molecular Medicine, Sant’Andrea University Hospital, Sapienza University, 00189 Rome, Italy; giacomo.tinimelato@uniroma1.it (G.T.); beatrice.musumeci@uniroma1.it (B.M.); emanuele.barbato@uniroma1.it (E.B.); 3 Arrhythmia Department, Teknon Heart Institute, Teknon Medical Center, 08022 Barcelona, Spain; giuliofalasconi@gmail.com (G.F.); penela.maceda@gmail.com (D.P.); antonio.berruezo@quironsalud.es (A.B.); 4 IRCCS Humanitas Research Hospital, 20089 Rozzano, Italy; 5 Division of Cardiology, Cardiovascular and Thoracic Department, “Citta Della Salute e Della Scienza” Hospital, 10126 Turin, Italy; 6 Cardiology Unit, Madre Giuseppina Vannini Hospital, 00177 Rome, Italy; luca.arcari88@gmail.com; 7 Department of Clinical Internal, Anesthesiological and Cardiovascular Sciences, Sapienza University, 00161 Rome, Italy

**Keywords:** hypertrophic cardiomyopathy, sudden cardiac death, ventricular tachycardia, ventricular fibrillation, substrates, cardiovascular magnetic resonance, electrophysiological study, afterdepolarizations

## Abstract

Sudden cardiac death (SCD), the most devastating complication of hypertrophic cardiomyopathy (HCM), is primarily triggered by ventricular tachycardia or fibrillation. Despite advances in knowledge, the mechanisms driving ventricular arrhythmia in HCM remain incompletely understood, stemming from an interplay of multiple pro-arrhythmic factors. Myocyte disarray and myocardial fibrosis form a structural substrate favorable to re-entrant arrhythmias by altering myocardial electrophysiological properties, while cellular abnormalities predominate in patients without evident structural remodeling. Traditional SCD risk prediction models rely on clinical risk factors and regression-based risk estimation, often overlooking specific arrhythmic substrates. Emerging techniques now allow for the direct assessment of these substrates, providing deeper insights into the arrhythmogenic mechanisms and paving the way for more personalized SCD risk stratification. This review explores the contribution of cellular, structural, and electrophysiological substrates to arrhythmic risk in HCM, emphasizing their distinct roles. Furthermore, it highlights the potential of substrate-based approaches to refining SCD prevention strategies and improving outcomes for patients with HCM.

## 1. Hypertrophic Cardiomyopathy and Sudden Cardiac Death: An Overview

Hypertrophic cardiomyopathy (HCM) is a common inherited myocardial disease, affecting approximately 1 in 500 individuals in the general population [1,2]. The hallmark of HCM is left ventricular (LV) hypertrophy in the absence of other cardiac or systemic conditions that cause hemodynamic overload [3]. Hypertrophy in HCM is typically asymmetric, mainly involving the basal anterior septum and the anterolateral free wall. However, the phenotypic expression is highly variable, and hypertrophy may symmetrically involve the entire LV or be confined to the free wall or apex [4].

HCM is inherited as an autosomal dominant trait with incomplete penetrance, and around 1400 causative mutations have been identified in 11 sarcomeric genes, with pathogenic variants in the MYH7 and MYBPC3 genes accounting for the majority of cases [5,6]. These mutations are identified in up to 60% of individuals with a family history of HCM and in approximately 30–40% of sporadic cases [7]. The onset of HCM occurs in three distinct life stages: infancy, adolescence, and middle adulthood [8]. Childhood-onset HCM is predominantly linked to sarcomeric mutations, whereas non-sarcomeric HCM is most often diagnosed after age 50, with a peak incidence in the sixth decade of life [8].

Initially, HCM was considered a disease with an exceptionally poor prognosis [9]. Advancements in diagnostic techniques and findings from studies on unselected populations have significantly reshaped our understanding of the natural history of the disease [10]. HCM is now recognized as having an extremely heterogeneous clinical course, ranging from asymptomatic presentation and stable course to catastrophic outcomes such as arrhythmic sudden cardiac death (SCD) and heart failure (HF) [11,12]. While most patients with HCM have a normal life expectancy, those with a complex clinical course may experience a variety of HCM-related complications [13]. Approximately 70% of patients develop LV outflow tract obstruction at some point, which may be present at rest or induced by exertion, affecting both symptoms and prognosis [1,14,15,16]. About 5% of patients progress to overt LV dysfunction and refractory HF with extensive fibrosis, resulting in a hypokinetic-dilated or restrictive phenotype, and often require transplantation (end-stage phase) [17,18]. Additionally, around 5% of patients develop an apical aneurysm, associated with a high risk of arrhythmic and thromboembolic events [19,20]. A small subset, approximately 10%, experience “benign remodeling”, characterized by gradual LV wall thinning with minimal changes in cavity dimension and ejection fraction, both of which remain within normal limits [21,22]. These patients generally have favorable long-term outcomes, with morbidity and mortality similar to those without LV remodeling [21]. The diverse phenotypic presentations underscore the importance of a personalized management strategy tailored to each patient’s distinct clinical profile, disease progression, and associated complications.

Among the complications associated with HCM, SCD is the most unpredictable and dramatic and may occur as the first disease manifestation. Together with HF and stroke, it contributes significantly to HCM-related mortality [23]. SCD in HCM is most commonly due to sustained ventricular tachycardia (VT) or ventricular fibrillation (VF) [24]. Contrary to earlier beliefs, SCD is relatively rare in HCM, with an annual incidence of about 0.7% in unselected populations [25]. SCD predominantly affects younger individuals, reaching a cumulative incidence of approximately 8–10% over 5 years in childhood [8]. The risk of SCD decreases with age and is low in HCM patients over 60 years old [26,27]. The prevention of SCD with implantable cardioverter defibrillators (ICDs) has transformed the natural history of HCM, reducing disease-related mortality more than tenfold and becoming a cornerstone of disease management [23,28]. However, ICD therapy is not without risks [29,30]. Thus, while significant progress has been made in developing defibrillation technologies with a lower risk of long-term complications [31,32,33,34], it is essential to refine risk stratification strategies, making them more effective and personalized.

Over the past few decades, SCD risk models have been developed and integrated into clinical guidelines, providing support in identifying candidates for ICD therapy [2,35]. However, catastrophic events still occur in patients classified as “low risk” [36], while many “high-risk” patients receive ICD implants but never require appropriate therapies [37], exposing them to lifelong ICD-related complications without any survival benefit [37]. The pro-arrhythmic mechanisms underlying ventricular arrhythmias in HCM are complex and not yet fully understood. They involve cellular anomalies, macro- and microstructural abnormalities, and changes in myocardial electrophysiological properties. Current prediction models for SCD primarily focus on clinical risk factors, such as a family history of SCD, left ventricular wall thickness, non-sustained VT, an unexplained syncope, and abnormal blood pressure responses to exercise [35]. While these models effectively support clinical decision-making in refining ICD candidacy, a significant proportion of patients who experience SCD do not meet the established high-risk criteria. This underscores the need for a more individualized approach to risk assessments.

Recent advancements in molecular studies, imaging, and electrophysiological techniques have significantly deepened our understanding of the structural and functional substrates underlying arrhythmic events in HCM. Unlike traditional reviews that primarily address established clinical risk factors, this work adopts a mechanistic perspective, analyzing the electrophysiological and pathophysiological basis of ventricular arrhythmias through the integration of advanced technological insights. By leveraging modern approaches to substrate characterization, this review envisions the transition from static risk stratification models to a dynamic, physiology-driven framework for SCD prediction. This paradigmatic shift has the potential to refine risk assessment and pave the way for a more precise and personalized approach to shared decision-making in ICD implantation.

The organization of this review into distinct sections—cellular/molecular, anatomical/structural, and electrophysiological/functional—reflects a methodological approach aimed at structuring the available evidence in a systematic manner. While these domains are inherently interconnected, particularly through the influence of cellular mechanisms on structural remodeling and functional alterations, this framework allows for a more defined analysis of the current state of knowledge and its implications.

## 2. Cellular Substrates

HCM has traditionally been considered a disease that primarily affects the myocardium, resulting in macro- and microstructural abnormalities [22]. Therefore, research has predominantly focused on structural substrates as the main drivers of ventricular arrhythmias. Despite advances in understanding pro-arrhythmic structural substrates, fatal events continue to occur, particularly in patients without significant structural abnormalities [38,39,40]. Notably, the most lethal arrhythmia occurs in young patients at the early stages of the disease, when structural remodeling is minimal and replacement fibrosis may be absent. This subset of patients remains one of the greatest challenges in understanding SCD in HCM.

A primary reason for our inadequate knowledge in this area is that limited translational research exists, while most efforts have focused on clinical markers of risk derived from pathology, diagnostic tool-based evaluations, and imaging [41]. Few studies have focused on the cellular mechanisms of arrhythmogenesis, which might explain the occurrence of arrhythmias in patients without manifest structural abnormalities, though not exclusively. In this subgroup of patients, sustained arrhythmias begin with one or more premature ventricular contractions (PVCs), arising from the abnormal spontaneous depolarization of clusters of synchronized ventricular myocytes and propagating through the LV [41]. It has been hypothesized that the arrhythmogenic substrate involves early (EADs) and delayed afterdepolarizations (DADs) related to ion current dysfunctions and altered intracellular calcium (Ca^2+^) homeostasis [42,43,44,45,46]. EADs occur during phases 2 or 3 of the cardiac action potential, typically when repolarization is prolonged. Patch-clamp studies on isolated HCM cardiomyocytes have shown that action potential duration (APD) is significantly prolonged compared to controls, resulting from multiple ion current abnormalities: a reduction in potassium (K^+^) currents (including inward-rectifier, transient outward, and delayed rectifier K^+^ currents), an increase the L-type Ca^2+^ current (ICaL), and a heightened late sodium (Na^+^) current (INaL) [43,47,48]. DADs, on the other hand, arise after the cell has fully repolarized (phase 4 of the cardiac action potential) due to intracellular Ca^2+^ overload. Calcium overload generates an inward sodium current through the Na^+^/Ca^2+^ exchanger, resulting in delayed depolarization [46,49,50]. Abnormal Ca^2+^ handling has consistently been observed in both animal models and human samples of HCM [43,47,49,51,52,53]. Alterations in Ca^2+^ homeostasis are driven by various concurrent mechanisms, including the sustained activation of CaMKII, the increased amplitude and delayed inactivation kinetics of ICaL, a reduced SERCA/phospholamban ratio, diminished SERCA expression, the loss and disorganization of t-tubules, increased Ca^2+^ leakage from the sarcoplasmic reticulum, and an abnormal Na^+^/Ca^2+^ exchanger function [42,43,44,45,46]. This collective cascade of mechanisms creates a vicious cycle, driving intracellular Ca^2+^ accumulation and promoting delayed depolarizations, further increasing the risk of arrhythmogenic events [43]. Adding to this complexity, an abnormal response to beta-adrenergic stimulation represents another potential cellular substrate for arrhythmogenesis in HCM, particularly during exercise or stress. A preclinical study showed that while beta-stimulation shortens APD in control cells, isoproterenol paradoxically prolongs APD in HCM cardiomyocytes, suggesting an abnormal cellular response under stress conditions [47]. Another factor to consider is the pro-arrhythmic role of myocardial wall stress mediated by the activation of stretch-activated channels (SACs). SACs are ion channels sensitive to mechanical stress that induce intracellular fluxes of calcium (Ca^2+^) and sodium (Na^+^), triggering afterdepolarizations [54]. Preclinical models have demonstrated that SAC activation can lead to electrophysiological instability and remodeling [54], with arrhythmogenic effects associated with SAC currents increasing with hypertrophy [55]. It is reasonable to hypothesize that the heightened myocardial wall stress typically observed in HCM could activate these channels, contributing to increased arrhythmic risk. However, these observations remain speculative, as no direct evidence currently supports a definitive role for SACs in the incidence of SCD in HCM.

Arrhythmogenesis due to cellular abnormalities is the hallmark of other inherited cardiac conditions associated with SCD that lack structural substrates, such as channelopathies [56]. Although concurrent mutations in both sarcomeric and ion channel genes have been observed in some patients [57], preclinical studies have identified a direct mechanistic link between mutant sarcomeric proteins (e.g., increased myofilament Ca^2+^ sensitivity) and cellular arrhythmogenicity [52,58]. Interestingly, arrhythmogenic cellular alterations were detected very early in transgenic mouse models before overt structural and functional cardiac abnormalities developed [42,59].

While these preclinical findings provide valuable pathophysiological insights, in vivo data remain limited. It has been extensively demonstrated that a family history of SCD is one of the most important risk factors, and it has been hypothesized that this is due to the transmission of proarrhythmic mutations, some of which may potentially involve cellular substrates [60,61,62,63]. Patients with sarcomere mutations are more prone to SCD and exhibit more pronounced repolarization abnormalities compared to those with a negative genetic status [64,65,66,67]. A recent prospective study showed that ventricular repolarization abnormalities are present in patients with HCM-related genetic mutations but without phenotypic expression [66]. Proarrhythmic abnormalities in the absence of scars or hypertrophy may reflect early cellular and molecular changes in response to sarcomeric mutations. These interesting findings should be interpreted with caution, keeping in mind that arrhythmogenesis in HCM is complex and involves multiple mechanisms. In fact, Diffusion Tensor Imaging (DTI) has shown that even in the absence of hypertrophy or fibrosis detectable with conventional imaging, microstructural abnormalities can be observed in the early stages of HCM and may contribute to the electrophysiological abnormalities observed [68].

While thought-provoking, the assessment of cellular substrates currently has minimal to no clinical applicability in identifying high-risk HCM patients. However, advanced ECG studies or drug challenges, similar to those used for channelopathies, may hold diagnostic and prognostic value in the near future [41]. Additionally, drugs targeting cellular pro-arrhythmic substrates could play a role in SCD prevention. Ranolazine, an INaL inhibitor, has been shown in preclinical studies to reduce APD and EADs in HCM cardiomyocytes [43,47]. It was also tested in a small multicenter, placebo-controlled, randomized trial, where it modestly reduced ventricular ectopy on a 24 h Holter ECG [69]. Similarly, disopyramide is a Na^+^ channel blocker that has been used for years in obstructive HCM for its negative inotropic properties and has been found to stabilize the closed state of the RyR2 channel, thereby inhibiting Ca^2+^-dependent arrhythmias and DADs by reducing cytosolic calcium levels [48]. Finally, novel cardiac myosin inhibitor drugs, which reversibly inhibit the myosin–actin coupling reaction, have the potential to reduce the calcium sensitivity of myocardial myosin and inhibit Ca^2+^-dependent arrhythmias [70]. Their preventive effect on ventricular arrhythmias and SCD has not been adequately investigated, requiring longitudinal data.

### Key Messages

- Cellular substrates play a critical role in the arrhythmogenic mechanisms of HCM, particularly in patients without manifest structural abnormalities.
- Ventricular arrhythmias and SCD can occur early in the disease, driven by ion current dysfunction, impaired calcium homeostasis, and abnormal beta-adrenergic responses. These proarrhythmic changes often precede overt structural remodeling and may be linked to sarcomeric mutations.
- Although cellular mechanisms currently have limited clinical applicability in identifying high-risk HCM patients, advancements in molecular diagnostics, imaging, and targeted therapies hold promise for improving SCD risk management.

## 3. Structural Substrates

Myocardial hypertrophy in HCM is characterized by a range of macroscopic and microscopic structural abnormalities [71]. In at least one-third of the myocardium, myocytes are hypertrophic and disorganized, displaying structural abnormalities in both shape and alignment, which is a phenomenon collectively referred to as myocardial disarray. This typically involves hypertrophic walls but may also affect areas of the ventricular wall with normal thickness [71]. In the HCM myocardium, the extracellular matrix is expanded and particularly rich in glycogen, with widespread interstitial fibrosis that can become so extensive as to cause progressive fibrotic replacement of the myocardium, leading to scar formation [72]. Furthermore, structural abnormalities of the intramyocardial coronary vessels are commonly observed, including media-layer hypertrophy, disorganized elastic fibers, and endothelial hyperplasia. These changes result in vessel wall thickening, which narrows the luminal diameter and leads to microvascular dysfunction, impairing the heart’s ability to increase myocardial perfusion in response to metabolic demands. The result is myocardial ischemia, myocyte necrosis, and the replacement of these cells with additional fibrotic tissue [73,74,75,76].

The combination of myocardial disarray, increased extracellular matrix, and microvascular abnormalities results in both micro- and macroscopic structural alterations, dominated by the formation of myocardial fibrosis. This scarring, in the form of interstitial or replacement fibrosis, is a recognized hallmark of HCM and is commonly found in the necropsies of young patients with SCD [77].

Myocardial scars are essential components of re-entry circuits, the most common electrophysiological substrate of ventricular arrhythmias [78]. A re-entry circuit requires an anatomical or functional barrier, at least two distinct pathways for the electrical impulse to travel down (one of which must have different conduction properties, such as slower conduction), a unidirectional block (the impulse cannot travel in one direction but can still travel in the opposite), and sufficient excitable tissue. When these conditions are met, electrical excitation follows a slow, circular path. The time required for the excitation wave to complete the circuit exceeds the effective refractory period of the myocytes, allowing for persistent, self-sustaining re-entry. This re-entry circuit then functions as a rapid pacemaker, depolarizing the ventricular myocardium and leading to sustained ventricular tachycardia [41].

Regions of non-conductive dense scarring or anatomical structures act as anatomical obstacles that block the propagation of electrical impulses [79,80]. Conversely, myocardial disarray and interstitial fibrosis disrupt the physiological homogeneity of electrical conduction, favoring anisotropic, slow conduction [81]. Indeed, collagen strands accumulating in the spaces between cardiomyocytes can form thick insulating sheaths, impeding transversal conduction while accelerating longitudinal conduction [82,83]. Additionally, activated fibroblasts influence myocardial electrical activity directly by forming gap junctions with cardiomyocytes [84]. Ultimately, local transient ischemia due to microvascular dysfunction further contributes to arrhythmogenesis by creating regions of partial depolarization and slowed conduction, which further promotes anisotropic conduction [77,85,86].

Cardiac magnetic resonance (CMR) is the gold-standard non-invasive tool for identifying and quantifying both replacement and interstitial fibrosis [87,88]. CMR detects replacement fibrosis by exploiting the distinct kinetics of paramagnetic contrast distribution in the healthy myocardium versus scar tissue (late gadolinium enhancement technique, LGE). Fibrosis appears as hyperintense signals, varying in intensity based on the contrast accumulated in the interstitial space, which reflects the scar architecture. Scar tissue can be qualitatively assessed and quantified [87]. More recent techniques, such as T1 mapping and extracellular volume fraction (ECV) measurement, enable the detection of interstitial fibrosis by evaluating its effects on the longitudinal magnetization recovery time [89]. Finally, DTI is an emerging CMR technique that visualizes myocardial microstructure by mapping water molecule diffusion and measuring disarray [90].

Early studies assessing structural substrates as predictors of arrhythmic risk in HCM focused on total scar burden, which has proven to be a strong predictor of adverse events in several unselected prospective cohorts [79,91,92,93]. The largest analysis exploring LGE as a risk factor for HCM-related SCD demonstrated that events peaked in patients with a high scar mass (≥15%), while no significant increase in SCD risk was observed in patients with minimal LGE (1–5%) compared to those without any LGE [79]. Two following prospective cohort studies demonstrated that q moderate LGE extent (10–15%) predicts major arrhythmic events in patients with low-to-intermediate SCD risk [94,95]. Consequently, the American College of Cardiology/American Heart Association (ACC/AHA) incorporated a scar burden threshold of 15% as a risk factor to consider in the subgroup of patients without major clinical SCD risk factors or when ICD implantation remains uncertain [2]. However, scar burden as a sole risk factor has limited sensitivity, as demonstrated by a high rate of adverse events in patients with LGE ≤ 15% [79,94,95].

**Figure 1:**
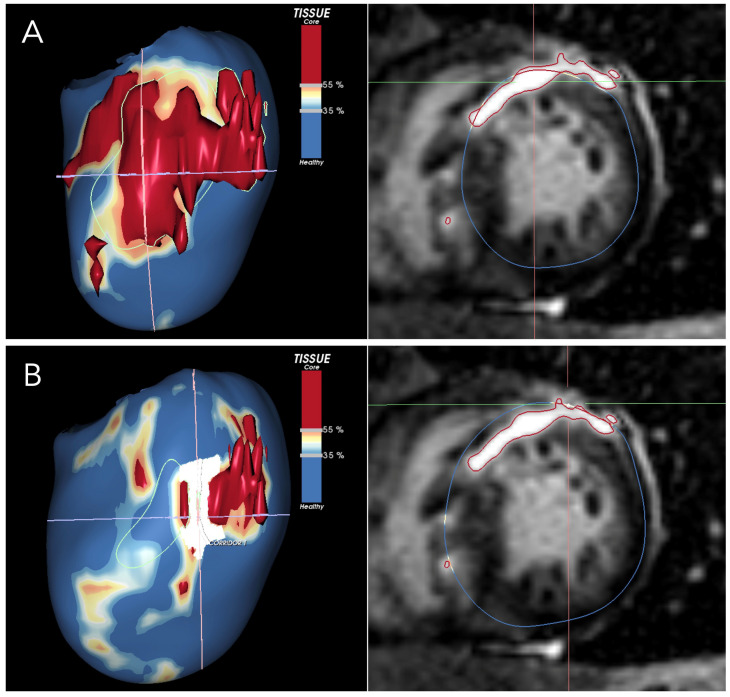
The figure shows a short-axis view of a CMR of a patient with HCM, highlighting an extensive scar involving the anterior wall and the interventricular septum. LGE-CMR images were post-processed using ADAS 3D (Galgo Medical, Barcelona, Spain), creating nine concentric surface layers spanning from the endocardium to the epicardium of the left ventricular wall thickness, resulting in a 3D shell for each layer. Color-coded pixel signal intensity (PSI) maps were projected onto each shell. Hyper-enhanced areas were classified as the core zone, borderline zone (BZ), or healthy tissue using thresholds of 40 ± 5% and 60 ± 5% of the maximum PSI. The scar-dense core is coded in red, BZ is coded in orange and white, and healthy myocardium is coded in blue. BZ channels are identified as continuous corridors of BZ protected by scar core tissue. In (**A**), the 50% layer is shown, representing the mid-myocardial layer. The area is entirely occupied by a dense and compact scar. In (**B**), the 3D reconstruction corresponds to the 80% epicardial layer. In the anterior wall, at the center of the crosshair, a BZ channel is visible.

LGE in HCM patients reveals variations in presentation, signal intensity, and distribution independently of global scar extent [96]. This heterogeneity reflects the complex architecture of HCM scar tissue, which, unlike post-ischemic scars, consists of diffuse fibrosis combined with viable muscle fibers rather than dense subendocardial scarring confined to a specific vascular territory [74,97,98,99]. This complex architecture represents an ideal substrate for re-entry circuits. CMR studies showed that mild- or intermediate-enhanced LGE was more predictive of ventricular arrhythmias than hyper-enhanced LGE [100,101]. Furthermore, a recent study in low- and intermediate-risk patients demonstrated that a quantitative analysis of LGE dispersion is a predictor of major arrhythmic events independently of the total scar burden [96]. Visual scar heterogeneity evaluation can be challenging and is prone to significant interobserver variability. Radiomics has the potential to make this analysis more accurate and reproducible. A recent study conducted on a large unselected cohort of HCM patients demonstrated that scar heterogeneity assessed with LGE radiomics is a significant predictor of SCD risk and provides incremental risk stratification beyond the current risk models [102]. This suggests that a qualitative assessment of scar architecture through LGE signal heterogeneity may provide more detailed insights into the pro-arrhythmic substrate, enabling the risk reclassification of patients with mild-to-moderate LGE burden (<15%). The main limitation of assessing LGE heterogeneity with standard LGE images is its reliance on 2D imaging, which does not fully capture the complex three-dimensional architecture of scar tissue [96]. Recently developed post-processing software elaborated LGE images to obtain a more accurate evaluation of myocardial scar architecture [103,104]. By segmenting pixel signal intensity, these techniques enable differentiation between dense core fibrosis and diffuse border zone (BZ) fibrosis. They then reconstruct the data into 3D images and identify corridors of BZ tissue protected by dense scars or anatomical barriers that connect areas of normal myocardium between the core zones, referred to as border zone channels (BZCs) (Figure 1) [103]. From a functional point of view, BZCs are corridors of excitable myocardial tissue with slow conduction, electrically insulated by non-conductive scar tissue. By acting as slow-conducting re-entrant pathways, they can trigger and sustain ventricular tachycardia [103]. In this view, BZC may represent the imaging equivalent of the structural and functional substrate that promotes re-entrant VTs [105]. A recent CMR study demonstrated that BZCs are a strong independent predictor of ventricular arrhythmias in high-risk HCM patients and that risk assessment based on scar channels rather than total scar mass reclassified approximately 12% of patients, aiding in the identification of those at highest risk [106].

As discussed, in addition to the identification of electrically inert scars, a substrate assessment model aimed at identifying potential reentrant circuits must also be capable of detecting areas of possible impaired conduction, including those with myocardial disarray. In this regard, cardiac DTI is an innovative CMR technique that measures water diffusion in three dimensions, allowing for the characterization of the myocardial microstructure [90]. By calculating fractional anisotropy (FA), cardiac DTI quantifies the directional variability of water diffusion. FA values near zero indicate random diffusion (perfect isotropy), while values near one indicate unidirectional diffusion (perfect anisotropy). Thus, FA is expected to be high in voxels with coherently aligned myocytes and low in voxels with differing myocyte orientations due to disarray [81]. A recent study found that disarray detected by cardiac DTI (as reduced FA) correlated with ventricular arrhythmia risk independently of the presence of fibrosis and hypertrophy [81]. These findings reinforce the concept that disarray is a critical factor in arrhythmogenesis, even in the very early stages of the disease [68].

### Key Message

- Myocardial scars and myocyte disarray are structural substrates with recognized, significant pro-arrhythmic implications in HCM. However, the assessment of myocardial disarray is still not technically reliable and is not yet included in the definition of arrhythmic risk.
- While LGE estimation is useful as a quantitative, non-binary variable, it seems pathophysiologically inadequate to explain the structural and functional complexity of the electrical substrates.
- The heterogeneity and complexity of scar architecture and disarray in HCM necessitate a more nuanced evaluation, which is now achievable through advanced imaging techniques. This refined approach could allow for more precise identification of pro-arrhythmic substrates and lay the foundation for substrate-based risk stratification.

## 4. Electrophysiological Substrates

Although the etiology of SCD in HCM is arrhythmic, the study of myocardial electrophysiological properties has traditionally played a marginal and controversial prognostic role in this condition [35]. Most spontaneous non-sustained ventricular arrhythmias in HCM are monomorphic, and a significant proportion of ICD interventions are triggered by monomorphic VTs [91,107,108,109,110,111]. However, polymorphic VT or VF has traditionally been regarded as the most common mode of arrhythmic death and is the most frequently induced form of arrhythmia during programmed ventricular stimulation (PVS) in these patients [112,113]. Monomorphic ventricular arrhythmias are likely related primarily to scar-mediated re-entry mechanisms, whereas the anatomical and functional substrates underlying polymorphic arrhythmia in HCM remain poorly understood.

Re-entry circuits typically encompass protected isthmuses of slow-conducting tissue [78]. Of note, myocardial disarray has been associated with regions of impaired conduction [114,115], although demonstrating its effects on electrical conduction in vivo remains challenging [116]. The Paced Electrogram Fractionation Analysis (PEFA) technique is an electrophysiological method designed to assess conduction heterogeneity and electrical activity within the myocardium [117,118,119,120]. This procedure involves pacing specific sites in the right ventricle (apex, mid-septum, RVOT, and free wall) with programmed stimuli and recording the resulting electrograms. Each site is paced (S1) with a decremental sequence, and an additional premature stimulus (S2) is introduced after every third beat, with recordings made from the remaining electrodes. The electrograms are then analyzed for duration, amplitude, and fractionation—characterized by fragmented potentials at the end of the electrogram—as an indicator of slow and heterogeneous conduction. This technique exposes the electrophysiological effects of disarray, including delayed intramyocardial conduction due to slowed or tortuous pathways and a temporary conduction block [120]. Spatially heterogeneous depolarization and repolarization are known to be pro-arrhythmic by favoring the asymmetric excitability and propagation of re-entry [116]. PEFA quantifies these effects through measurements of electrogram duration and the S1-S2 coupling interval at which delays increase. Prospective studies have shown PEFA to predict major arrhythmic events with greater accuracy than non-invasive techniques [117,118,120]. Interestingly, recordings made in sinus rhythm did not show the same alterations as those observed with pacing and associated with SCD [116]. This suggests that the arrhythmogenic electrophysiological substrate is dynamic, with the myocardial perturbation that triggers arrhythmia being mimicked by pacing [116].

Scar tissue, along with disarray, is the critical structural substrate for re-entrant arrhythmias, and invasive electrophysiological studies can detect scar-induced electrophysiological alterations [79,121,122]. The extent of myocardial scarring correlates with the inducibility of ventricular arrhythmias during PVS [123]. Additionally, direct recordings from bipolar electrograms on the endocardial surface of LGE segments in HCM show low amplitude and prolonged signals, suggesting conduction delay [122]. These findings indicate the presence of the slow-conducting viable myocardium within scarred areas, creating an ideal environment for sustaining re-entrant VTs and functionally confirming what has been observed with 3D CMR imaging techniques. These regions of viable myocardial tissue within the BZ, surrounded by non-conductive scar tissue, may act as slow-conducting re-entrant pathways that sustain VT circuits. Supporting this concept, a prospective cohort study demonstrated that the presence of BZ channels correlates with the inducibility and characteristics of ventricular arrhythmias during PVS [124]. In this study, polymorphic VT or VF induced by PVS was often preceded by a short run of monomorphic VT originating from within the scar. This suggests that the arrhythmia may begin as monomorphic, utilizing re-entry circuits with BZ channels as critical components, and subsequently degenerate into disorganized arrhythmia due to the sarcomeric misalignment and non-uniform anisotropic conduction characteristic of the HCM myocardium, which favors a high degree of activation dispersion, slow conduction, and unidirectional blocks [124]. These findings derive from a single, monocentric study and require confirmation by further research; however, they offer novel insights into the pathophysiology of ventricular arrhythmias in HCM.

**Figure 2:**
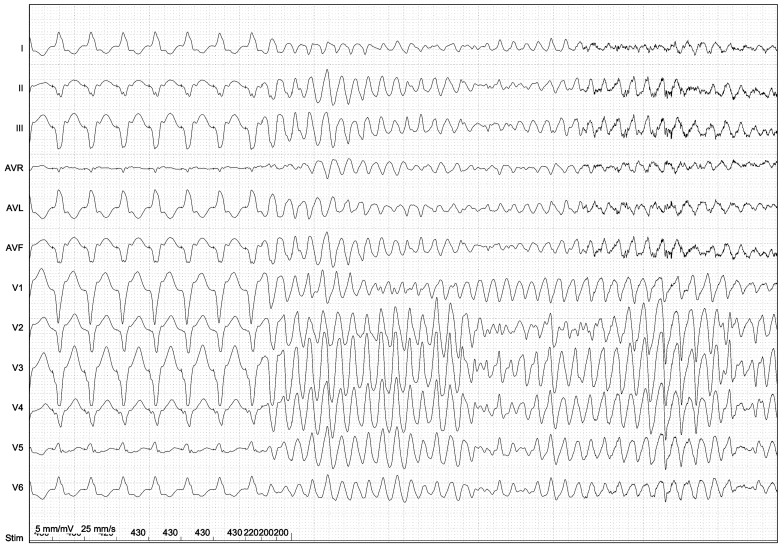
Induction of ventricular fibrillation by programmed ventricular stimulation (PVS) in a patient with HCM. A basic drive train cycle length of 430 ms is followed by three extra stimuli with short coupling intervals (220, 200, and 200 ms) applied in the right ventricle. The prognostic significance of polymorphic VT and VF induced with PVS in HCM is unknown. Aggressive stimulation protocols may lack specificity when identifying true arrhythmic risk.

The role of PVS has long been controversial in HCM. For many years, PVS was considered non-specific and lacking prognostic value beyond the non-invasive risk factors, leading to its very limited use in contemporary HCM risk stratification (Figure 2) [2,35,125,126]. However, earlier studies suggested that PVS could provide prognostic information and guide therapy in specific low-risk subgroups [112]. A single-center prospective study in a cohort of unselected patients recently found that inducibility at PVS predicted SCD or appropriate device therapy in HCM, while non-inducibility correlated with prolonged event-free survival [127]. The study reported an inducibility rate of ~ 39%, with a positive predictive value of approximately 23%. This inducibility rate was notably lower than in previous studies (67–77%), likely due to differences in patient selection and the use of less aggressive stimulation protocols [126]. Indeed, the high inducibility rate due to aggressive PVS has previously led to this prognostic tool being regarded as having limited specificity [125,128]. The main limitation of PVS is that it investigates induced arrhythmias rather than spontaneous ones. As such, there is no sufficient evidence to fully rely on invasive electrophysiological studies as the sole factor for arrhythmic risk stratification in HCM. However, while further investigation is needed to confirm its prognostic accuracy, these new findings re-evaluate PVS and suggest its potential application in specific patient subgroups.

The heterogeneity of spontaneous ventricular repolarization assessed with a 12-lead ECG in HCM has been associated with CMR-detected fibrosis and has been shown to be a prognostic factor in arrhythmic risk stratification [129,130]. However, 12-lead ECG techniques quantifying depolarization and repolarization abnormalities have seen limited clinical use due to challenges related to spatial resolution, technical limitations, and bias toward more advanced disease stages [131,132,133,134]. New non-invasive techniques have been developed to map ventricular activation and repolarization with high spatial and temporal resolution [135,136,137,138,139,140]. A recent prospective study using an integrated non-invasive electrophysiological approach combined with advanced cardiovascular imaging (CMR-guided electrocardiographic imaging, ECGI) demonstrated correlations between structural substrates and functional electrophysiological alterations [66]. Moreover, the translational potential of computational modeling also offers promising paths for advancing arrhythmia management in HCM. Indeed, emerging clinical applications of digital twins [141] promise to revolutionize care by providing dynamic, personalized insights into electrophysiological behavior. If these techniques continue to evolve to become more accessible, cost-effective, and reproducible, future longitudinal studies may reassess the role of electrophysiological substrates and redefine current approaches.

### Key Messages

- Structural substrates have functional electrophysiological counterparts that are gradually emerging as crucial in defining the mechanisms of ventricular arrhythmia in HCM patients.
- These findings open promising future perspectives for an integrated imaging–electrophysiological approach, complementing clinical risk factors for a more comprehensive and personalized risk assessment.

Table 1 summarizes the substrates of SCD in HCM. 

## 5. Conclusions

SCD is the most unpredictable and severe complication of HCM. Traditional SCD prediction models regress the clinical risk factors against events to estimate risk, with limited consideration of the underlying arrhythmic substrates. While these models have significant value in clinical practice, they also have limitations. They excel at identifying high-risk patients with advanced disease but are less effective for those in the early stages of disease, with minimal remodeling or subtle anatomical and functional substrates. The advent of new techniques could enable direct assessment of arrhythmic substrates, providing a more detailed understanding of the mechanisms involved. In the near future, these advancements may facilitate a more targeted and personalized approach to SCD risk assessment in HCM, shifting the paradigm from general clinical risk predictors to an individualized, substrate-based evaluation.

## Figures and Tables

**Table 1:** Substrates of sudden cardiac death in hypertrophic cardiomyopathy.

Substrate	Pathological Abnormalities	Functional Alterations	Arrhythmia	Supporting Evidence
Cellular	Ion currents dysfunction: Reduction in K^+^ currents Increased ICaL current Increased InaL	APD prolongation → EADs Ca^2+^ overload → DADs Abnormal response to beta-adrenergic stimulation	Premature ventricular contractions Polymorphic VT/VF	Coppini R. et al. Circulation 2013 [43] Olivotto I. et al. Circ Heart Fail 2018 [69]
	Abnormal intracellular Ca^2+^ homeostasis			
Structural and Electrophysiological	Replacement fibrosis Interstitial fibrosis Myocardial disarray Microvascular ischemia	Conduction block Slow conduction	Reentry arrhythmia	Chan RH. et al. Circulation 2014 [79] Francia P. et al. JACC Cardiovasc Imaging 2023 [106] Saumarez RC et al. Circulation 1992 [117]

ICaL: L-type Ca^2+^ current. InaL: late sodium current. APD: action potential duration. EADs: early afterdepolarizations. DADs: delayed afterdepolarizations.
